# The Role of PTHLH in Ovarian Follicle Selection, Its Transcriptional Regulation and Genetic Effects on Egg Laying Traits in Hens

**DOI:** 10.3389/fgene.2019.00430

**Published:** 2019-05-14

**Authors:** Xiaoli Guo, Yiya Wang, Qiuyue Chen, Zhenjie Yuan, Yuxia Chen, Miao Guo, Li Kang, Yi Sun, Yunliang Jiang

**Affiliations:** ^1^Shandong Provincial Key Laboratory of Animal Biotechnology and Disease Control and Prevention, College of Animal Science and Veterinary Medicine, Shandong Agricultural University, Tai’an, China; ^2^College of Life Science, Qilu Normal University, Jinan, China

**Keywords:** hen, PTHLH, follicle selection, promoter, egg laying traits, AP-1

## Abstract

In hens, follicle selection is an important process affecting egg laying traits. This study investigated the role of parathyroid hormone-like hormone (PTHLH) in chicken follicle selection, its transcriptional regulation and genetic effects on egg laying traits. PTHLH and its receptor PTH1R were mainly expressed in follicles of 6–8 mm in diameter, exhibits differential expression pattern in the theca and granulosa cells of pre- and hierarchal follicles. PTHLH stimulates the proliferation of follicular granulosa and theca cells, the expression of StAR and CYP11A1 mRNA and the production of progesterone (P4) in pre-hierarchal follicles. Treatment with FSH increased PTHLH mRNA expression in pre-hierarchal follicular theca cells and hierarchal follicular granulosa cells. Two critical regions regulating chicken PTHLH transcription were revealed, each of which harbored a SNP: C>T (chr1: 72530014) for AP-1 and a SNP: A>G (chr1: 72531676). Hens with diplotype AC/GT were younger at first laying and laid more eggs at 32 weeks. The haplotype (G^-1827^T^-165^) with double mutations had the greatest promoter activity of chicken PTHLH transcription. Collectively, PTHLH plays an important role in chicken follicle selection by stimulating cell proliferation and steroidogenesis. Polymorphisms in chicken PTHLH promoter region are associated with egg laying traits by affecting the binding of transcription factor AP-1.

## Introduction

Parathyroid hormone-like hormone (PTHLH), also called parathyroid hormone-related protein (PTHrP), was initially discovered in 1987 because of its causative role in the humoral hypercalcemia of malignancy (Moseley et al., 1987). It is evolutionarily related to parathyroid hormone (PTH) and belongs to a family of endocrine factors that share a highly conserved N-terminal region (Potts, 2005; Guerreiro et al., 2007). PTHLH plays multiple roles in regulating calcium transport (Weir et al., 1996; Miao et al., 2004; Martin, 2005), bone formation and skeletal development (Cuthbertson et al., 1999; Zhao et al., 2002; Klopocki et al., 2010), and smooth muscle tension (Thiede et al., 1990; Ferguson et al., 1994). It also regulates cell growth and differentiation in a broad variety of cell types and organs, including pancreatic islet (Vasavada et al., 1996), kidney (Aya et al., 1999) and breast (Wojcik et al., 1998; Luparello et al., 2003), and cellular events like endocytosis (Aarts et al., 1999a; Pache et al., 2006) and nucleolar translocation (Massfelder et al., 1997; Aarts et al., 1999b).

Parathyroid hormone-like hormone and PTH share common receptors, but different subsets of the PTH/PTHLH receptor (PTHR) are used by different vertebrate species; for example, mammals have two PTH receptor genes, PTH1R and PTH2R (Abou-Samra et al., 1992), but chicken lacks a PTH2 gene and its receptor PTH2R, and uses only PTH1R as its receptor (Bhattacharya et al., 2011; Li et al., 2014). PTH1R is a highly conserved G-protein coupled receptor that is activated nearly equivalently by PTH and PTHLH (Juppner et al., 1991; Gardella and Juppner, 2001), widely expressed in both fetal and adult tissues, and is essential for the normal regulation of systemic mineral ion homeostasis (Fan et al., 2016).

A laying hen contains many different ovarian follicles that range in size from 1 to 40 mm, and were grouped according to size: 1–2, 3–5, 6–8, and 9–12 mm (the most recently selected), and the smallest hierarchal follicles F6 to the largest one F1 (Tilly et al., 1991b; Woods and Johnson, 2005; Liu and Zhang, 2008; Lin et al., 2011). In regularly laying hens, a single follicle among the small cohort of follicles of 6–8 mm in diameter is daily selected into the hierarchy to enter the rapid growth phase and undergo final maturation before ovulation, the process of which is called follicle selection that is directly related to egg laying traits (Liu and Zhang, 2008). A widespread distribution and expression of PTHLH were reported in chicken bone, cartilage, skin, and kidney tissues (Pinheiro et al., 2010), however, the role of PTHLH in the process of follicle selection remains unknown.

Our preliminary analysis on the transcription profile of chicken follicles of 6–8 mm in diameter revealed an upregulation of PTHLH gene, along with the increase of follicle stimulating hormone receptor (FSHR) mRNA expression (Wang et al., 2017), suggesting that PTHLH likely plays some roles in follicle selection. Therefore, in this study, the function of chicken PTHLH in the process of follicle selection, the mechanism of its transcriptional regulation and its genetic effect on egg laying traits in hens were investigated. The results indicated that PTHLH plays important roles in follicle selection, is regulated by FSH and has positive effect on egg laying traits in hens.

## Materials and Methods

### Animals and Sample Collections

Hy-line brown hens 35–40 weeks of age and been laying regularly for at least 1 month were used in this study. Hens were housed under standard conditions with free access to food and water. All sampled hens were killed by cervical dislocation immediately after oviposition. Follicles at different developmental stages were collected and immediately frozen in liquid nitrogen.

Hens from five chicken breeds (lines), including White Recessive Rock (*n* = 550), Hy-line brown (*n* = 45), Wenchang (*n* = 50), Jining Bairi (*n* = 50), Xinyang brown (*n* = 50), were randomly sampled from their respective breeding populations and used for polymorphism analysis. The egg laying traits of age at first egg (AFE) and the total number of eggs at 32 weeks (E32) of the White Recessive Rock population were recorded individually for association analysis. The White Recessive Rock hens were pure line, and reared on the same farm with the same feeding conditions. All hens had free access to water and feed. Genomic DNA was extracted from blood samples collected from the wing vein using a DNA Extraction mini kit (Tiangen Biotech, Beijing, China).

The Institutional Animal Care and Use Ethics Committee of Shandong Agricultural University reviewed and approved all procedures described in this study. This study was performed in accordance with the “Guidelines for Experimental Animals” of the Ministry of Science and Technology of China.

### 5′- and 3′-RACE

Total RNA was isolated from chicken ovarian follicles using a Total RNA Kit (Tiangen Biotech, Beijing, China), assessed using a spectrophotometer (Eppendorf BioPhotometer plus, Hamburg, Germany) and checked by loading total RNA onto a 1% agarose gel that was stained with ethidium bromide. To create the full-length mRNA, gene-specific primers for 5′- and 3′-RACE were designed with a 262 bp overlap, and nested primers with a 48 bp overlap. ASMARTer^TM^ RACE cDNA Amplification Kit (Clontech, United States) was used, and the 5′- and 3′-sequences of chicken PTHLH cDNA were obtained by cloning and sequencing the RACE products. Gene-specific primers and nested primers were shown in Supplementary Table S1.

### Culture and Treatment of Follicular Theca and Granulosa Cells

Follicles were divided into pre-hierarchal (6–8 mm) and hierarchal follicles. Pre-hierarchal follicles were treated with 0.1% collagenase II (MP Biomedicals, Santa Ana, CA, United States) at 37°C for 8 min to disperse pre-hierarchal follicular granulosa cells and for an additional 30 min to disperse pre-hierarchal follicular theca cells. Theca cell and granulosa cell layers from each hierarchal follicle were collected and combined within their respective group and then dispersed for culture as previously described (Gilbert et al., 1977). Hierarchal follicular granulosa cell layers were dispersed by treated with pancreatin (Gibco, Camarillo, CA, United States) for 15 min, while theca cell layers were dispersed with collagenase II for 30 min. The isolated theca and granulosa cells were planted in a 24-well culture plate containing 1 mL of M199 complete medium with high glucose (Gibco, Camarillo, CA, United States) plus 10% fetal bovine serum (Biological Industries, Israel). The cells were then cultured with serum-free M199 medium in the absence or presence of different concentration of rhFSH (Sigma, St. Louis, MO, United States) for 24 h. The RNA from the cultured cells was isolated with a MicroElute Total RNA Kit (Omega Bio-tek, Norcross, GA, United States).

### Real-Time Quantitative RT-PCR

The cDNA was synthesized using a Primescript^TM^ RT reagent Kit with gDNA Eraser (TaKaRa, Dalian, China), and the resultant cDNA was stored at -20°C for mRNA expression analysis. Real-Time quantitative PCR (qRT-PCR) was conducted on an MX3000p instrument (Stratagene, La Jolla, CA, United States) using the SYBR premix ExTaq (TaKaRa, Dalian, China). Melting curves were used to confirm the specificity of each product, and the PCR efficiencies were determined by analysis of twofold serial dilutions of cDNA that were designed to detect all the signals in the spanning region. The efficiencies were nearly 100%, and therefore, the 2^-ΔΔCT^ method for calculating the relative gene expression levels was used (Livak and Schmittgen, 2001; Zhu et al., 2012). The β-actin gene was used as the internal control, and the primer sequences were shown in Supplementary Table S1.

### Western Blotting

Crude proteins were extracted from different developing follicles or the four types of cells, which were homogenized in cell lysis buffer containing the protease inhibitor PMSF (Beyotime, Beijing, China). A BCA Protein Assay Kit (Novoprotein, Shanghai, China) was used to determine protein concentration. An equal amount of protein was separated by 10% gel electrophoresis under denaturing and non-reducing conditions using an SDS-PAGE Gel Quick Preparation Kit (ComWin Biotech, Beijing, China). Then, the proteins were transferred to a 0.22 μm polyvinylidene fluoride membrane (Solarbio, Beijing, China). The immunoblot analysis was performed using rabbit anti-chicken PTHLH polyclonal antibody (Abbiotec, San Diego, CA, United States) or PTH1R polyclonal antibody (Genscript, NJ, United States) at a dilution of 1:500, and a mouse polyclonal anti-β-actin IgG (1:750) was used for standardization. Incubations with primary antibodies were conducted overnight at 4°C with gentle agitation. The horseradish peroxidase-conjugated goat anti-rabbit/mouse IgG secondary antibody (1:1000) was incubated for 2 h at room temperature. Peroxidase activity was detected with a BeyoECL Plus Kit (Beyotime, Beijing, China). Then ImageJ software was used to quantify the expression level of PTHLH and PTH1R protein.

### Immunohistochemistry

Routine serial sections of formaldehyde-fixed, paraffin-embedded tissue blocks of the follicles were cut into 4-μm-thick slices and then deparaffinized with xylene and rehydrated through a graded ethanol series. After pressure cooking in 10 mM sodium citrate buffer, the sections were incubated in 3% hydrogen peroxide for 15 min to block endogenous peroxidase activity, and incubated in 10% goat serum for 15 min prior to incubation with the rabbit anti-chicken PTHLH or PTH1R polyclonal IgG antibody (1:100) for 2.5 h at 37°C. The sections were then incubated with the secondary antibody for 30 min and the avidin-biotin-peroxidase complex for another 30 min according to the Histostain-plus kit instructions (Zhongshan Golden Bridge Biotechnology, Beijing, China). Finally, the immunoprecipitates were visualized using a diaminobenzidine (DAB) kit (Zhongshan Golden Bridge Biotechnology, Beijing, China). The slides were counter stained with hematoxylin after immunostaining, dehydration and covering. Negative controls were prepared in each case by replacing the primary antibody with PBS; no specific staining was found in the control slides.

### Overexpression and Knockdown Assay

The entire coding region of chicken PTHLH gene was amplified using forward primers containing the *Mlu*I site and a reverse primer containing the *Hin*dIII site (the primers were shown in Supplementary Table S1), and polymerase PrimeSTAR (TaKaRa, Dalian, China) was used to ensure high fidelity. The PCR fragments were generated by double enzyme digestion and ligated with pUSEamp(+) expression vectors (Upstate, NY, United States) by T4 DNA ligase, which were transformed into DH5α competent cells. After being confirmed by bidirectional sequencing and purified using an EndoFree Plasmid Purification Kit (Qiagen, Valencia, CA, United States), these plasmids were transfected into cells. Empty pUSEamp(+) vector was used as the control.

The three siRNAs were designed according to the chicken PTHLH mRNA sequence (GenBank accession number NM_001174106.1). The negative control of the siRNA had the same composition with the siRNA sequence but had no homology with PTHLH mRNA (Shanghai GenePharma Co., Shanghai, China). Negative control and siRNAs (the sequence are shown in Supplementary Table S1) were diluted and used according to the instructions, and the most effective siRNA was used to analyze the knockdown effect of siRNA on chicken PTHLH gene.

### Progesterone Radioimmunoassay

Progesterone levels in media samples were quantified using an Iodine[^125^I]-Progesterone Radioimmunoassay Kit (XieHe, Tianjin, China). Briefly, tubes with 100 μl standards in different concentrations, quality control serum or samples were numbered and added 200 μl ^125^I-progesterone marker, then 200 μl of antibody was added to each tube except T and NSB tubes, mixed well and incubated for 1 h at 37°C. One milliliter separation agent was added to each tube and stored 10 min at room temperature, centrifuged for 20 min at 3500 rpm, then the supernate was aspirated and CPM was counted with a gamma counter RIA program for 1 min. The standard curve was established by B/B0 of each standard and its concentration, and the concentration of progesterone was read from the standard curve. The minimum detection concentration for progesterone was 0.05 ng/mL and the intraassay coefficiency was 0.9999.

### Cell Transfection and Cell Proliferation Assay

The pUSE-PTHLH plasmid or siRNA was transfected when cells were grown to 80% confluency using NanoFectin Transfection Reagent (Shanghai ExCell Biology, Shanghai, China). Twenty-four hours later, the cells were cultured with serum-free M199 medium for another 24 h.

Proliferation of cells was quantified by MTT staining (Berridge and Tan, 1993) at 0, 24, 48, and 72 h after cell transfection using an MTT Cell Proliferation and Cytotoxicity Assay Kit (Beyotime, Beijing, China). The MTT solution (5 mg/mL) that was assessed and incubated for 4 h at 37°C was added to formazan lysis buffer for a further 4 h incubation until all the formazan dissolved. Cell number was evaluated using an ELx808 Absorbance Reader at 570 nm.

### Promoter Deletion Analysis and Luciferase Assay

The region from -3574 to +98 bp in the 5′-regulatory region of chicken PTHLH promoter, and six promoter deletion fragments, -2989/+98, -2046/+98, -1428/+98, -1380/+98, -611/+98, and -526/+98, were amplified from hen genomic DNA, where +1 is the transcription initiation site. Seven forward primers contain the *Nhe*I site at 5′ ends, and one reverse primer located downstream to the transcription start site contains the *Kpn*I site at 5′ ends (primer sequences are listed in Supplementary Table S1). All PCR fragments were digested with *Nhe*I and *Kpn*I restriction enzymes and ligated with pGL3-Basic vector (Promega, Madison, WI, United States).

Four plasmids including wt-PTHLH (A^-1827^C^-165^/A^-1827^C^-165^), mut-TF1 (A^-1827^T^-165^/A^-1827^T^-165^), mut-TF2 (G^-1827^C^-165^/G^-1827^C^-165^), and mut-PTHLH (G^-1827^T^-165^/G^-1827^T^-165^) were constructed to assess the functionalities of two transcription factor binding sites in PTHLH promoter in response to FSH stimulation. The wt-PTHLH and mut-PTHLH constructs were constructed in the same way with the -2046/+98 deletion construct, with individuals of A^-1827^C^-165^/A^-1827^C^-165^ wild genotype and G^-1827^T^-165^/G^-1827^T^-165^ mutant genotype as a template, respectively. The single SNP was mutated using a Fast Site-Directed Mutagenesis Kit (Tiangen Biotech, Beijing, China), the mut-TF1 was the mutation of the -165 site from TGACcCT to TGACtCT, and mut-TF2 was the mutation of the -1827 site from ATGGaG to ATGGgG. The primer oligonucleotide sequences were shown in Supplementary Table S1. All plasmids were bidirectionally sequenced and purified as described above.

The pGL 4.74 control vector (Promega, Madison, WI, United States) and different-sized PTHLH promoter luciferase reporter plasmids or wild and mutation plasmids were transfected with the same method as the overexpression plasmid. Luciferase activity was measured using the dual-luciferase reporter assay system according to the manufacturer’s protocol (Promega, Madison, WI, United States). The enzymatic activity of luciferase was measured with a luminometer (Modulus TM, Turner Biosystems). The transfections were performed at least in triplicate, and the individual values were averaged for each experiment. Empty pGL3-basic was used as the control.

### SNP Identification, Polymorphism, and Association Analysis

Initially, 48 individuals from each line were used as temples for PCR amplification to determine the critical promoter region of PTHLH; then, eight individuals per pool, six pools per line, were sequenced. The data sequenced bidirectionally were analyzed using the DNAMAN program to determine the number of potential SNPs within these lines. Primer pairs were the same with the -2046/+98 deletion construct (the primers are shown in Supplementary Table S1). The genotype at the -1827 SNP site was determined by forward sequencing and at the -165 SNP site by reverse sequencing. Linkage analysis and haplotypes construction were analyzed using SHEsis software^1^. The genotype and allele frequencies and Hardy-Weinberg equilibrium *P*-value were calculated using the Tools for Population Genetic Analyses software^2^. The associations of PTHLH diplotypes with egg laying traits in the White Recessive Rock hen populations were analyzed using the following general linear model of the SAS statistical software package (version 9.2; Cary, NC, United States):Y_ij_ = m + G_i_ + e_ij_, where Y_ij_ is the phenotypic value of traits, μ is the population mean, G_i_ is the fixed effect of genotype, and e_ij_ is the random error effect. The putative transcription initiation site and transcription factor binding sites were predicted using AliBaba2.1^3^ and MatInspector^4^.

### Electrophoretic Mobility Shift Assay (EMSA)

Mo7e cells were seeded at a density of 1 × 10^6^ cells/mL and incubated in IMDM with 10% FBS at 37°C for 72 h with TNF-α (negative control with albumin). The nuclear extracts prepared from the cells were incubated with biotin-labeled double-stranded oligonucleotides containing the consensus sequences for NF-κB (Wt-NFκB: 5′-CGCATCTGTATGGgGATGAAGGGAG-3′; Mut-NFκB: 5′-CGCATCTGTATGGaGATGAAGGGAG-3′) and AP-1 (Wt-AP1: 5′-CGCTCCCACTGACtCTGCCGCCCCT-3′; Mut-AP1: 5′-CGCTCCCACTGACcCTGCCGCCCCT-3′) for an additional 4 h. EMSA was performed using Non-Radioactive EMSA Kits with Biotin-Probes (Viagene, Tampa, FL, United States). The DNA-protein complex and unbound probe were electrophoresed on a 6% native polyacrylamide gel and visualized as for the western blotting. AP-1 or NF-κB monoclonal antibody was used for the super shift, and standard NF-κB was used as positive control.

### Data Analysis

Experiments were repeated a minimum of three independent times using tissues from different hens. Real-time PCR results were expressed as fold differences compared with an appropriate control tissue or treatment. Difference between the experimental groups were evaluated with ANOVA, followed by Duncan’s multiple range test (*p* < 0.05), using the general linear model procedure of the SAS statistical software package.

## Results

### Expression of PTHLH and Its Receptor PTH1R in Chicken Ovarian Follicles

**FIGURE 1:**
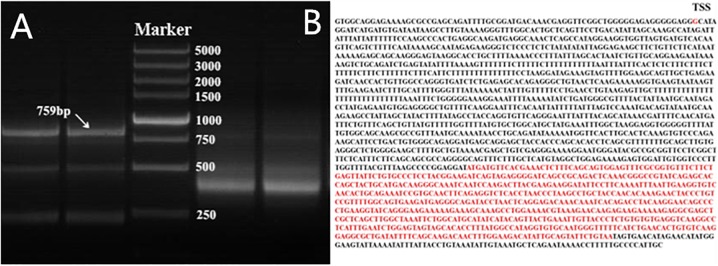
Nested PCR results of 5′- and 3′-RACE of chicken PTHLH gene. **(A)** 5′- and 3′-RACE products were separated by 1% agarose gel electrophoresis. 3′-RACE product is on the left of Marker, and 5′-RACE product is on the right of Marker. DL 5,000 DNA Marker (TaKaRa, Dalian, China) consisted of eight fragments between 250 and 5,000 bp. **(B)** The mRNA sequence of PTHLH transcript variant X1, the transcription start site (TSS) was G, and the CDS has been marked with red color.

Because ten transcripts of the chicken PTHLH gene are reported in chicken tissues (GenBank accession number NC_006088.4), we first determined the one expressed in the chicken ovarian follicles by 5′- and 3′-RACE. After sequencing and assembly of the RACE products, the main transcript expressed in chicken ovarian follicles was 759 bp in size, which is the transcript variant 1 in NCBI (GenBank accession number NM_001174106.1) (Figure 1). Therefore, the following experiment was performed based on this transcript.

**FIGURE 2:**
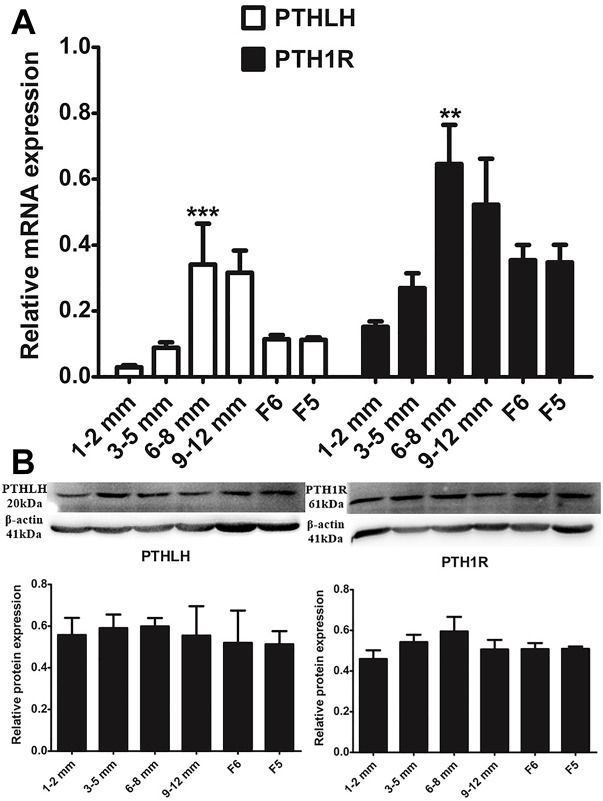
PTHLH and PTH1R expression in different chicken follicles. **(A)** mRNA expression of chicken PTHLH and PTH1R genes was analyzed by real-time PCR. **(B)** Protein expression of chicken PTHLH and PTH1R was analyzed by Western blot analysis. All data are presented as the means ± SEM. ^∗∗^*p* < 0.01, ^∗∗∗^*p* ≤ 0.001.

With the increase in follicular volume, the mRNA expression of both PTHLH and PTH1R gradually increased, reaching the highest level in follicles of 6–8 mm in diameter; expression then slightly decreased in follicles of 9–12 mm in diameter and markedly decreased in F6 and F5 follicles (*p* ≤ 0.001 for PTHLH; *p* ≤ 0.001 for PTH1R). The protein expression dynamics of PTHLH and PTH1R in different follicles were somewhat higher in follicles of 6–8 mm in diameter than in other follicles; however, the difference was not statistically significant (*p* > 0.05) (Figure 2).

### Expression of PTHLH and PTH1R in the Granulosa and Theca Cells of Chicken Ovarian Follicles

**FIGURE 3:**
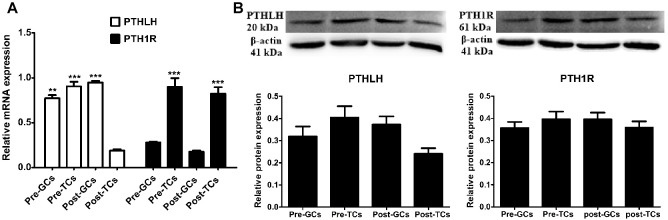
PTHLH and PTH1R expression in different types of chicken follicular cells. Expression of PTHLH and PTH1R mRNA **(A)** and protein **(B)** in the theca cells (TCs) and granulosa cells (GCs) of chicken follicles. All data are presented as the means ± SEM. ^∗∗^*p* < 0.01, ^∗∗∗^*p* ≤ 0.001.

We further compared the expression of mRNA and protein of chicken PTHLH and PTH1R genes in the granulosa and theca cells of pre- (6–8 mm) and hierarchal (F6-F1) follicles, which were designated as pre-GCs, post-GCs, pre-TCs, and post-TCs, respectively. Chicken PTHLH mRNA was expressed in both pre-GCs and pre-TCs, with expression significantly higher in pre-TCs than in pre-GCs (*p* < 0.05). After follicle selection, PTHLH mRNA was primarily detected in granulosa cells. The expression of chicken PTH1R mRNA was significantly higher in both pre- and hierarchal follicular theca cells than that in either type of granulosa cell (*p* < 0.001). However, the levels of protein expression were not significantly different among the four types of chicken follicular cells (*p* > 0.05) (Figure 3).

### Immunohistochemical Localization of PTHLH and PTH1R Proteins in Chicken Ovarian Follicles

**FIGURE 4:**
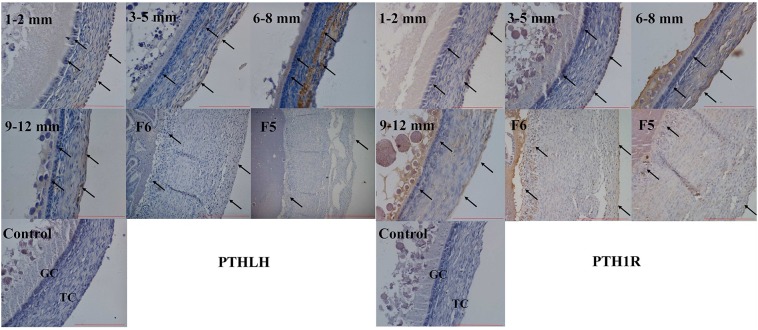
Localization of chicken PTHLH and PTH1R protein in the theca cells (TCs) and granulosa cells (GCs) of chicken follicles around follicle selection. Negative control with PBS used in place of primary antibody. Arrowheads indicate the position of stained protein. Redlines indicate 100 μm.

The localization of chicken PTHLH and PTH1R protein in pre-hierarchal and hierarchal follicles was examined by immunohistochemical method. Strong staining of PTHLH is in the theca cells of 6–8 mm follicles and that of PTH1R is in the theca and granulosa cells of 6–8 and 9–12 mm follicles. In F6 and F5 follicles, weak staining signal of PTHLH was detected, whereas still strong signal was detected for PTH1R in follicles around follicle selection, the chicken PTHLH and PTH1R proteins were localized in both granulosa cells and theca cells, with strong staining observed in follicles of 6–8 mm in diameter (Figure 4).

### Effects of PTHLH on the Proliferation of Chicken Follicular Cells

**FIGURE 5:**
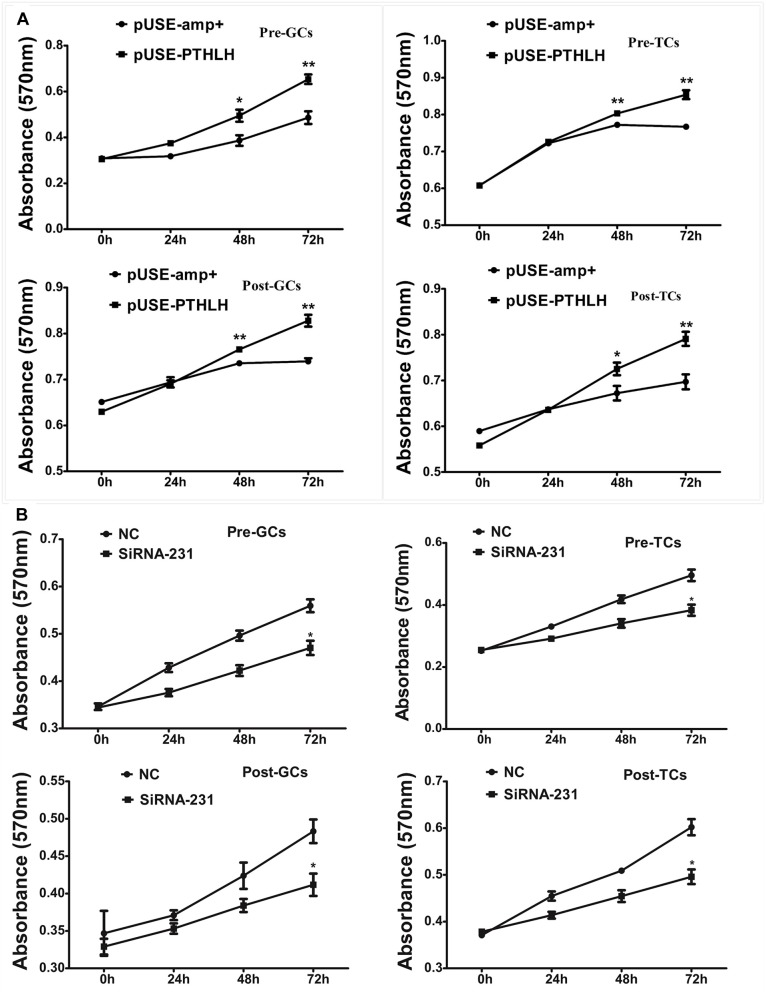
Effect of PTHLH on the proliferation of granulosa and theca cells from both pre- and hierarchal follicles. **(A)** Overexpression of PTHLH stimulated, and **(B)** knockdown of PTHLH inhibited, the proliferation of granulosa and theca cells isolated from both pre- and hierarchal follicles, respectively. All data presented as the means ± SEM. ^∗^*p* < 0.05, ^∗∗^*p* < 0.01.

Overexpression of chicken PTHLH gene stimulated the proliferation of not only granulosa cells but also theca cells collected from both pre-hierarchal and hierarchal follicles (Figure 5A; *p* < 0.05), and knockdown of PTHLH expression by small interfering RNA, i.e., siRNA-231, inhibited their proliferation (Figure 5B; *p* < 0.05).

### Effect of PTHLH on Steroid Hormone Synthesis in Pre-hierarchal Granulosa and Theca Cells

**FIGURE 6:**
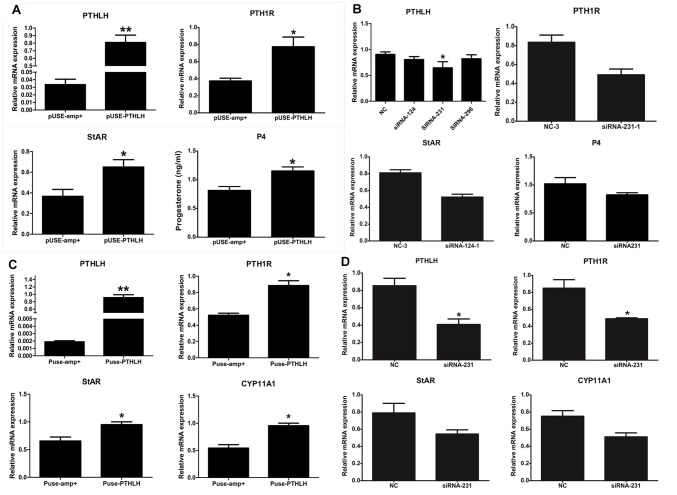
Effect of PTHLH on mRNA expression of steroidogenic enzyme genes and P4 production in chicken pre-hierarchal follicular cells. PTHLH promoted the mRNA expression of PTH1R and StAR and production of P4 in pre-GCs **(A)**, and **(B)** knockdown of PTHLH inhibited the mRNA expression of PTH1R and StAR and production of P4 in pre-GCs. **(C)** Overexpression of PTHLH increased the mRNA expression of PTH1R, StAR and CYP11A1 in theca cells isolated from pre-hierarchal follicles, while knockdown of PTHLH inhibited their expression **(D)**. All data are presented as the means ± SEM. ^∗^*p* < 0.05, ^∗∗^*p* < 0.01.

In pre-GCs, the mRNA expression of PTH1R, StAR, and the production of P4 was significantly increased when chicken PTHLH is overexpressed (Figure 6A; *p* < 0.05) (Supplementary Figure S1). On the contrary, knockdown of chicken PTHLH expression by RNAi (Supplementary Figure S2) decreased the expression of chicken PTH1R and StAR mRNA and the production of P4 in pre-GCs (Figure 6B; *p* < 0.05). In pre-TCs, overexpression of chicken PTHLH significantly increased the mRNA expression of chicken PTH1R in addition to that of StAR and CYP11A1, the two essential steroidogenic enzymes responsible for steroid synthesis (Figure 6C; *p* < 0.05), while knockdown of chicken PTHLH gene by RNAi reduced the expression of PTH1R (*p* < 0.05) and tended to decrease that of StAR and CYP11A1 (Figure 6D; *p* > 0.05).

### Effect of FSH Treatment on Chicken PTHLH and PTH1R Expression

**FIGURE 7:**
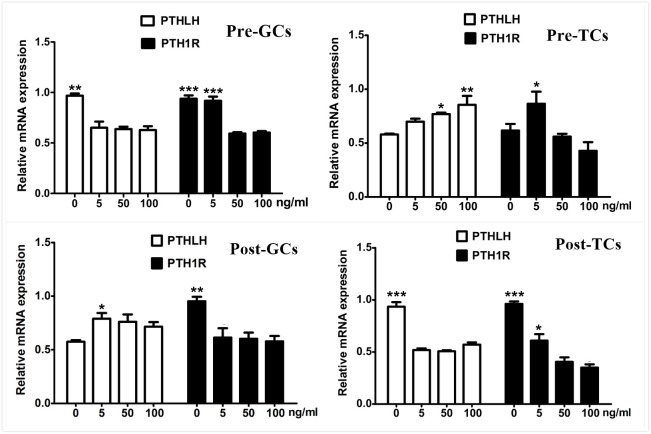
Effect of FSH treatment on PTHLH and PTH1R mRNA in granulosa and theca cells of chicken ovarian follicles. Different doses of FSH (0, 5, 50, and 100 ng/mL) on PTHLH and PTH1R mRNA expression in granulosa and theca cells of chicken ovarian follicles. All data are presented as the means ± SEM. ^∗^*p* < 0.05, ^∗∗^*p* < 0.01, ^∗∗∗^*p* ≤ 0.001.

As the elevated expression of PTHLH is accompanied along with that of FSHR, the effect of FSH on the expression of chicken PTHLH and PTH1R genes was analyzed. We found that, after FSH treatment, the mRNA expression of PTHLH was significantly decreased in granulosa cells of pre-hierarchal follicles, but increased in hierarchal follicles; however, that of PTH1R was significantly decreased in both pre- and hierarchal follicles. As for theca cells, after FSH treatment, the mRNA expression of PTHLH was increased in pre-hierarchal follicles, but decreased in hierarchal follicles; and that of PTH1R was increased in pre-hierarchal follicles after treatment with FSH at 5 ng/mL, but decreased in hierarchal follicles (Figure 7).

### Promoter Activity of Chicken PTHLH Gene

**FIGURE 8:**
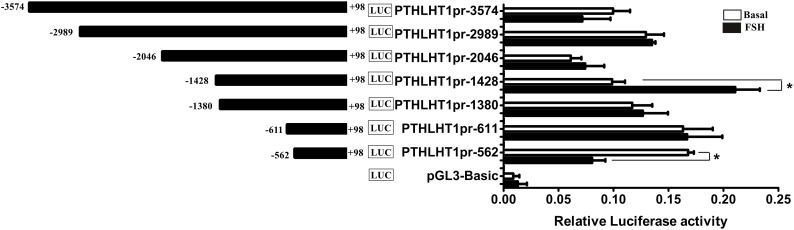
Promoter analysis of chicken PTHLH gene and the effects of FSH on PTHLH promoter activities in pre-TCs. The numbering refers to the transcription initiation site designated as +1. For deletion constructs of the chicken PTHLH promoter and an internal control vector pGL 4.74, luciferase activity was calculated by dividing the firefly luciferase activity by the Renilla luciferase activity. Each bar represents the means ± SEM. ^∗^*p* < 0.05 versus basal.

Because of the obvious effect of the chicken PTHLH gene on the proliferation and differentiation of follicular cells, the mechanisms regulating its transcription were analyzed. Two critical promoter regions were identified by promoter deletion: the region -1428 to +98 had the greatest response to FSH (10 ng/mL) stimulation, and FSH significantly decreased the promoter activity of the fragment -562 to +98 (Figure 8; *p* < 0.05). These results indicated that these two regions, -2046 to +98 and -562 to +98, may contain *cis*-acting response elements to FSH to regulate the transcription of chicken PTHLH gene.

### Polymorphisms in the Critical Promoter Region of Chicken PTHLH Gene

**FIGURE 9:**
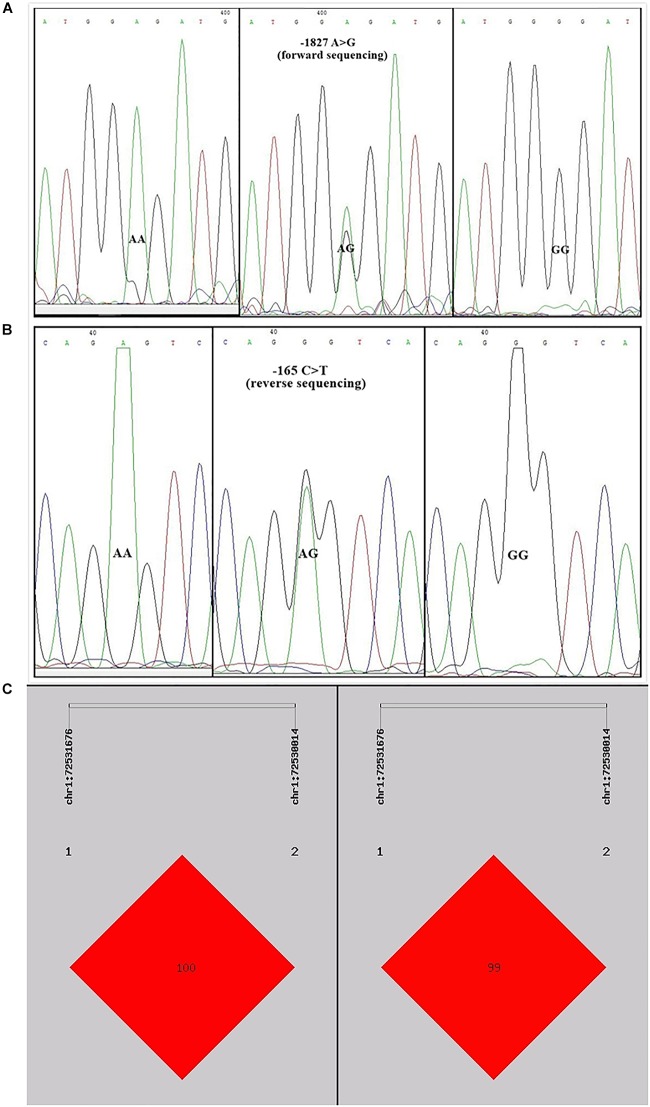
Two single nucleotide polymorphisms identified in the promoter region of chicken PTHLH gene **(A)** and the linkage disequilibrium (LD) result based on the online software SHEsis analysis in the White Recessive Rock population **(B,C)**. Normalized LD is shown as D’ **(B)** and R2 **(C)**; the values of D’ and R2 are in the range between 0 and 100%. D’ is a frequency-independent metric, and R2 is a measure of frequency. D’ and R2 value are 100% when the chain is in complete LD.

Sequence alignment between the promoter regions of chicken PTHLH gene from five chicken breeds (or lines) showed that each of the two critical promoter regions contains a SNP: rs72518862 (A>G) at -1827 and rs72520524 (C>T) at -165. Additionally, the two SNPs were in close linkage in the White Recessive Rock chicken population (Figure 9). The two SNPs had different genotype frequencies and allele frequencies in the five chicken populations. At SNP g.-1827 A>G, allele G was predominant in the Hy-line Brown, Xinyang Brown, and Jining Bairi populations, while allele A was predominant in the White Recessive Rock population, and at this SNP site, the allelic distribution deviated from Hardy-Weinberg equilibrium in the Jining Bairi population (*p* < 0.05). At SNP g.-165 C>T, allele C was predominant in the White Recessive Rock population, while allele T was predominant in the Hy-line Brown and Jining Bairi populations (Table 1).

**Table 1:** Genotype and allele frequencies of two SNPs of the chicken PTHLH gene in five chicken breed or lines.

SNP	Breed or line (number)	Genotype frequency			Allele frequency		HWE (*P*-value)
		AA	AG	GG	A	G	
g.-1827 A>G	White Recessive Rock (*n* = 550)	0.476	0.431	0.093	0.69	0.31	0.8046
	Hy-line Brown (*n* = 45)	0.044	0.289	0.667	0.19	0.81	0.7011
	Xinyang Brown (*n* = 50)	0.020	0.340	0.640	0.19	0.81	0.4595
	Wenshang Barred (*n* = 50)	0.280	0.400	0.320	0.48	0.52	0.1600
	Jining Bairi (*n* = 50)	0.040	0.580	0.380	0.33	0.67	0.0276

		**CC**	**CT**	**TT**	**C**	**T**	

g.-165 C>T	White Recessive Rock (*n* = 550)	0.476	0.431	0.093	0.69	0.31	0.8046
	Hy-line Brown (*n* = 45)	0.088	0.356	0.556	0.27	0.73	0.5420
	Xinyang Brown (*n* = 50)	0.160	0.600	0.240	0.46	0.54	0.1419
	Wenshang Barred (*n* = 50)	0.300	0.460	0.240	0.53	0.47	0.5877
	Jining Bairi (*n* = 50)	0.100	0.580	0.320	0.39	0.61	0.1215


### Association of Diplotypes of Chicken PTHLH Gene With Egg Laying Traits

Haplotype analysis revealed three haplotypes, i.e., AC, GC, and GT, in five chicken breeds or lines. Haplotype AC was predominant in the White Recessive Rock chicken population, while haplotype GT was predominant in the Hy-line Brown, Xinyang Brown, and Jining Bairi populations (Table 2). For the diplotypes AC/AC, AC/GT, and GT/GT, the association between diplotypes of the chicken PTHLH gene and egg laying traits was analyzed in White Recessive Rock chicken population. The results indicated that for hens with diplotype AC/GT, compared with the other diplotypes, the age at first laying was earlier and more eggs were laid at 32 weeks (*p* < 0.01) (Table 3).

**Table 2:** Haplotype frequency at the two SNP sites of the PTHLH gene in five chicken breeds (lines).

Breed or line (number)	Haplotype frequency			
	AC	AT	GC	GT
White Recessive Rock (*n* = 550)	0.692	0	0.002	0.306
Hy-line Brown (*n* = 45)	0.189	0	0.078	0.733
Xinyang Brown (*n* = 50)	0.19	0	0.27	0.54
Wenshang Barred (*n* = 50)	0.48	0	0.05	0.47
Jining Bairi (*n* = 50)	0.33	0	0.06	0.61


**Table 3:** Effect of the diplotypes of the two SNPs on laying traits in the White Recessive Rock chicken population.

Traits	Diplotypes			*P*-value
	AC/AC (*n* = 262)	AC/GT (*n* = 237)	GT/GT (*n* = 51)	
AFE	179.16 ± 0.48 A	176.46 ± 0.54 B	178.98 ± 1.34 A	0.0008
32E	33.42 ± 0.38 B	35.80 ± 0.47 A	33.27 ± 1.00 B	0.0002


### Effect of SNPs on Promoter Activity of Chicken PTHLH Gene

**FIGURE 10:**
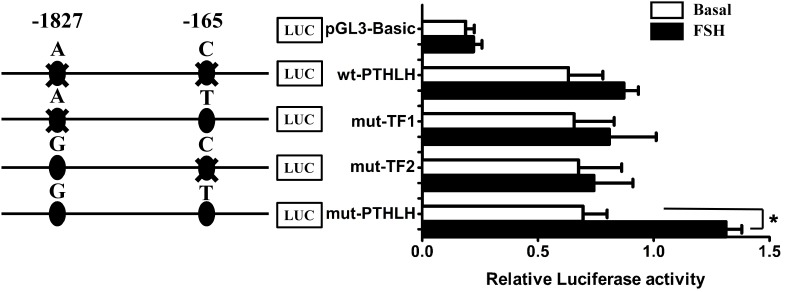
The activity of chicken PTHLH promoter harboring haplotype G^-1827^T^-165^ (mut-PTHLH) at the two SNPs (g.–1827 A>G and g. –165 C>T) increased after FSH treatment in chicken pre-hierarchal theca cells. All promoter constructs were co-transfected with an internal control vector pGL 4.74 into pre-hierarchal theca cells, and luciferase activities were measured after FSH (black bar) or vehicle (white bar) treatment. ^∗^*p* < 0.05 for differences between the corresponding constructs.

Luciferase reporter constructs of Wt-PTHLH (AC/AC), mut-TF1 (AT/AT), mut-TF2 (GC/GC), and mut-PTHLH (GT/GT) were transiently transfected into pre-TCs to assess whether the two SNPs could change the effect of PTHLH gene transcription activated by FSH. As shown in Figure 10, a single mutation of either SNP did not increase either the basal luciferase activity of the promoter or the induction of promoter activity by FSH; however, mutation of both SNPs significantly induced the promoter activity when stimulated by FSH (*p* < 0.01).

### Identification of Transcription Factor Binding Site at the Two SNPs in Chicken PTHLH Promoter

**FIGURE 11:**
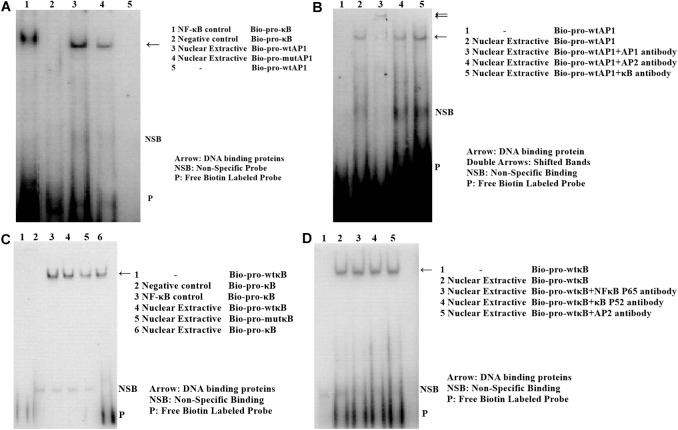
Electrophoretic mobility shift assay (EMSA) of the two transcription factor binding sites at two SNPs in chicken PTHLH promoter. EMSA analyses were conducted with wild and mutation biotin-labeled probe in SNP rs72520524 (C>T) **(A)** and SNP rs72518862 (A>G) **(C)**. Super shift assay was performed with AP-1 monoclonal antibody **(B)** and with NF-κB monoclonal antibody **(D)**.

Analysis with the AliBaba2.1 and MatInspector programs revealed that the SNP chr1: 72530014 (C>T) may alter the binding site of putative transcription factor AP-1 and that the SNP chr1: 72531676 (A>G) may change the binding site of putative transcription factor NF-κB. Electrophoretic mobility shift assay (EMSA) was performed to identify the two transcription factors. As shown in Figure 11, the change in SNP chr1: 72530014 from C to T increased the binding activity (Figure 11A), and the binding activity also increased with the change in SNP chr1: 72531676 from A to G (Figure 11C). Super shift assay with AP-1 monoclonal antibody appeared to correspond to the DNA-protein-antibody complex (Figure 11B), which further demonstrated that the SNP chr1: 72530014 was indeed located in the binding site of AP-1; however, the SNP chr1: 72531676 (A>G) was not contained in the binding site of NF-κB (Figure 11D).

## Discussion

The ovary of a laying hen has a lot of follicles, but only a very small number of them could mature and ovulate. In the process of follicle selection, only a single follicle from a cohort of follicles of 6–8 mm in diameter is selected to enter the pre-ovulatory hierarchy, and this follicle undergoes a rapid growth phase accompanied by an increase both in follicle size and yellow yolk deposition, until the final maturation and ovulation. This cyclic selection of a follicle depends on circulating FSH and the expression of FSHR (Tilly et al., 1991a; Webb et al., 2003; Ocon-Grove et al., 2012; Kim et al., 2013; Johnson et al., 2015; Johnson and Lee, 2016). Among all follicles of 6–8 mm in diameter, the one that expresses an elevated level of FSHR will be selected to enter the hierarchy, whereas the relative expression in others is similar (Woods and Johnson, 2005). Since PTHLH performs an active role in regulating cell growth and differentiation of mammals, and chicken follicle selection is a process involving proliferation of theca and granulosa cells and differentiation of granulosa cells, we investigated the role of PTHLH in follicle selection and its impact on egg laying.

In this study, PTHLH and PTH1R were found to be expressed in follicles of all sizes in hens. The expression of PTHLH and PTH1R were higher in the small yellow follicles than in other sized follicles. In chicken pre-hierarchal follicles, the expression of PTHLH mRNA was higher in TCs than in GCs, while it was the opposite in hierarchal follicles; the expression of PTHLH on protein level exhibits similar trend. For the expression of PTH1R, it is significantly higher in GCs than in TCs on mRNA level, while no obvious changes were detected on protein level (Figure 3), suggesting that the expression of PTH1R is regulated at translational level. The alterations in the expression of chicken PTHLH from pre- to hierarchal follicles suggest a transition of PTHLH expression from theca cells to granulosa cells occurred during follicle selection. Similar to the situation in mammals, FSH is proposed to be essential not only for associated cell differentiation following follicle selection (Johnson and Lee, 2016), but also for the steroidogenesis (Tilly et al., 1991b) in chicken follicles. In this study, FSH increased the expression of chicken PTHLH mRNA in pre-TCs and post-GCs, which subsequently stimulated cell proliferation and pre-GCs differentiation, implying that during chicken follicle selection, at least in some part, FSH regulates follicle development through the action of PTHLH.

In hens, granulosa cells from pre-hierarchal follicles are prososed to be in an undifferentiated state due to the inhibitory actions of mitogen-activated protein kinase (MAPK) and the suppression of FSHR signaling via cAMP (Tilly et al., 1991a). The proliferation of theca and granulosa cells and subsequent differentiation of granulosa cells are important events of follicle selection in hens. In this study, we found that the overexpression of chicken PTHLH stimulated cell proliferation of both GCs and TCs from pre-hierarchal and hierarchal follicles, while knockdown of PTHLH expression produced the opposite effect, supporting the hypothesis that PTHLH is an inducer of cells proliferation. One hallmark of chicken granulosa cell differentiation is the responsiveness of granulosa cell layers to FSH, which is measured by their capacity to produce steroids such as StAR and P4 (You et al., 1996). Here, we found that the overexpression of chicken PTHLH induced the mRNA expression of StAR and P4 production, while knockdown PTHLH inhibited their expression or production, suggesting that PTHLH likely stimulated granulosa cell differentiation.

**FIGURE 12:**
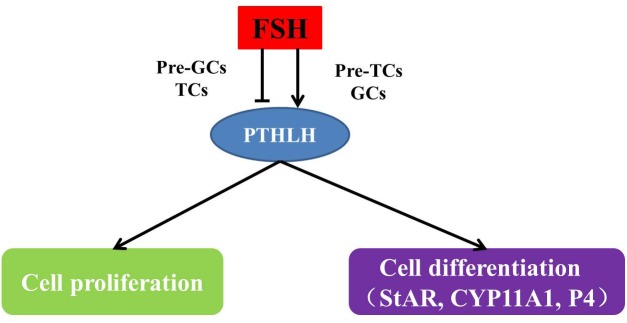
The mechanism of how PTHLH participates in chicken follicle selection.

During follicle selection in hens, with the stimulation of FSH and expression of StAR (Johnson et al., 2002) and CYP11A (Rangel et al., 2009), the differentiation of granulosa cells initiates in accompany with steroid synthesis. The production of steroids in ovarian follicles likely occurs through a cooperative interaction between the adjacent granulosa and theca layers. In pre-hierarchal follicles of the ovary, theca cells are the predominant source of steroid production, while a transition occurs in follicles of 6–8 mm in diameter under the stimulatory regulation of FSH and granulosa cells become steroidogenically competent (Porter et al., 1989; Tilly et al., 1991b). The granulosa cells produce progesterone, which is used by the theca interna to produce androgens, and subsequently metabolized to estrogens by the theca externa (Kim and Johnson, 2016). Steroidogenic incompetency in differentiating granulosa cells of pre-hierarchal follicles is primarily due to a lack of StAR and CYP11A (Tilly et al., 1991a; Johnson et al., 2002). Based on the results from this study, the overexpression of chicken PTHLH increased the expression of StAR and CYP11A1, while knockdown of its expression has some inhibitory effect, suggesting that PTHLH plays some stimulatory roles in steroidogenesis and follicle selection (Figure 12).

As the expression of chicken PTHLH varies around follicle selection, we analyzed the regulatory mechanism of PTHLH transcription and identified two critical regions, each of which contained a SNP (-1827 A>G and -165 C>T). Association analysis between the two SNPs and egg laying traits in the White Recessive Rock population showed that in hens with diplotype AC/GT, the age at first laying was younger and more eggs were laid at 32 weeks compared with the other diplotypes, implying that, at these two SNP sites, dominant effect is the main cause affecting egg laying traits. We identified one AP-1 binding site that contained SNP chr1: 72530014 (C>T) and another transcription factor (not NF-κB) binding site that contained chr1: 72531676 (A>G). Transcription factors AP-1 is critical in the regulation of cell proliferation and differentiation, and the most common component member of AP-1 is the c-Jun/c-Fos heterodimer, which upon activation binds to the TPA (PMA)-response elements in the promoter of various genes and usually works in combination with other transcription factors (Kwon et al., 2012; Lu et al., 2012; Khan et al., 2013, 2014; Choi et al., 2014; Limtrakul et al., 2016). In this study, the mutation of either SNP did not alter either the basal luciferase activity of the promoter or the promoter activity inducted by FSH. However, mutation of both SNPs significantly induced the promoter activity upon FSH stimulation. In this context, AP-1 likely works in combination with another yet unknown transcription factor(s) to mediate the regulation of chicken PTHLH transcription by FSH, which requires further study.

## Conclusion

In conclusion, the role of PTHLH in chicken follicle selection, its transcriptional regulation and genetic effects on egg laying traits were revealed in this study. PTHLH exhibits differential expression pattern in the theca and granulosa cells of pre- and hierarchal follicles and plays stimulatory roles in chicken follicular cell proliferation and differentiation around follicle selection. The two SNPs in the chicken PTHLH promoter region were associated with the traits of age at first egg laying and egg production at 32 weeks because of the alterations in two binding sites, including that of AP-1.

## Ethics Statement

The Institutional Animal Care and Use Ethics Committee of Shandong Agricultural University reviewed and approved all procedures described in this study. This study was performed in accordance with the “Guidelines for Experimental Animals” of the Ministry of Science and Technology of China.

## Author Contributions

XG, YW, and YJ contributed to the overall design of the manuscript. XG and QC collected the data. YW, QC, ZY, YC, and MG contributed to sampling process. YW, YJ, YS, and LK participated in manuscript writing and revision. All authors approved the final version of the manuscript.

## Conflict of Interest Statement

The authors declare that the research was conducted in the absence of any commercial or financial relationships that could be construed as a potential conflict of interest.

## Footnotes

1 http://analysis.bio-x.cn

2 http://www.marksgeneticsoftware.net/tfpga.htm

3 http://www.biobase-international.com/gene-regulation

4 http://www.genomatix.de
