# Supplementary material for: The Role of PTHLH in Ovarian Follicle Selection, Its Transcriptional Regulation and Genetic Effects on Egg Laying Traits in Hens
Source: Front Genet. 2019 May 14;10:430. doi: 10.3389/fgene.2019.00430 (PMC6530352; doi:10.3389/fgene.2019.00430)
Supplement: Supplementary file 1 [file Data_Sheet_1.docx]

Supplementary Material

The Role of PTHLH in Ovarian Follicle Selection, Its Transcriptional Regulation and Genetic Effects on Egg Laying Traits in Hens

Xiaoli Guo*, Yiya Wang*, Zhenjie Yuan, Yuxia Chen, Miao Guo, Li Kang, Yi Sun and Yunliang Jiang

*** Correspondence:** Yunliang Jiang: [yljiang723@aliyun.com](mailto:yljiang723@aliyun.com)

**Supplementary TABLE 1** The primers used in the experiments

| Experiments | Gene Symbol | Accession Number (GenBank) | Primer Sequence |
| --- | --- | --- | --- |
| Real-time PCR | PTHLH | NM_001174106 | Forward: 5’ AGAGGAACTGCGACGAACA  Reverse:5’ GGATTGATTTGCCCTTGTC |
|  | PTH1R | NM_001177575 | Forward: 5’ CCATAACTGAAGCACCTC  Reverse:5’ TGTGGAGATAGAGCCCTT |
|  | StAR | NM_204686 | Forward: 5’ TGCCTGAGCAGCAGGGATTTATCA  Reverse:5’ TGGTTGATGATGGTCTTTGGCAGC |
|  | CYP11A | NM_001001756 | Forward: 5’ ACTTCAAGGGACTGAGCTTTGGGT  Reverse:5’ AGTTCTCCAGGATGTGCATGAGGA |
|  | ACTB | NM_205518 | Forward: 5’ TGGATGATGATATTGCTGC  Reverse:5’ ATCTTCTCCATATCATCCC |
| Overexpression  5′-and 3′- RACE  SiRNA | PTHLH  PTHLH  PTHLH | NM_001174106  NM_001174106 | Forward: 5’ CGGCTAGCAGAGGAACTGCGACGAACAA  Reverse:5’ GGGGTACCTCAGCGCCTCCTTAAAATA  3'-RACE(F) TTCTGAGTTATTCTGTGCCCTCCTACGG  5'-RACE(R) TCTGCCCTCATCTTCACTGCCAA  Nest-3'(F) TCTCACCTAACCCTAAGCCTGCTACC  Nest-5'(R) CGGACAGGGTAGTTCTTTGTGTTGG  Negative control (sense)UUCUCCGAACGUGUCACGUTT  Negative control (antisense)ACGUGACACGUUCGGAGAATT  PTHLH-gga-231 (sense)GCAAUCAAUCCAAGACUUTT  PTHLH-gga-231 (antisense)AAGUCUUGGAUUGAUUUGCTT |


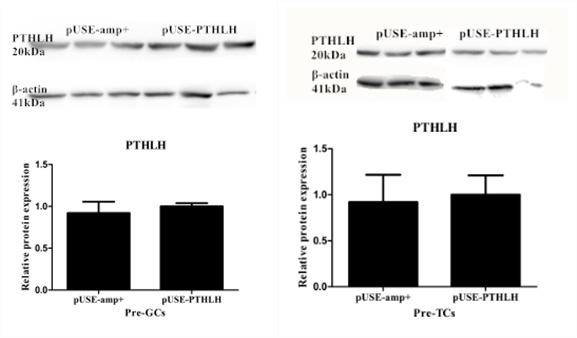


**Supplementary FIGURE 1** Expression of PTHLH protein in the Pre-GCs and Pre-TCs of chicken follicles when PTHLH is overexpressed.All data are presented as the means±SEM.


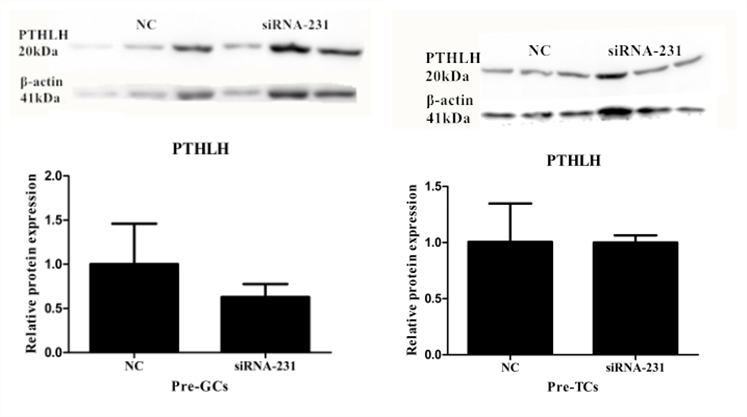


**Supplementary FIGURE 2** Expression of PTHLH protein in the Pre-GCs and Pre-TCs of chicken follicles when siRNA PTHLH is knocked down by using siRNA.All data are presented as the means±SEM.
